# Metabolic syndrome with mortality and major adverse cardiovascular events in an elderly population

**DOI:** 10.3389/fendo.2025.1570191

**Published:** 2025-05-22

**Authors:** Shailendra Kumar Karn, Fang Zhao, Yawei Xu, Yi Zhang, Chunlai Zeng, Imran Ibrahim Shaikh, Shekhar Singh, Yuling Feng

**Affiliations:** ^1^ Department of Cardiology, Shanghai Tenth People’s Hospital, Tongji University School of Medicine, Shanghai, China; ^2^ Department of Geriatrics, Shanghai Putuo People’s Hospital, School of Medicine, Tongji University, Shanghai, China; ^3^ Department of Cardiology, Central Laboratory of Lishui Hospital of Wenzhou Medical University, The First Affiliated Hospital of Lishui University, Lishui People’s Hospital, Lishui, Zhejiang, China; ^4^ Department of pharmacy, Central Laboratory of The Lishui Hospital of Wenzhou Medical University, The First Affiliated Hospital of Lishui University, Lishui, Zhejiang, China; ^5^ Department of Vascular Surgery, Lishui People’s Hospital, Lishui Hospital of Wenzhou Medical University, The First Affiliated Hospital of Lishui University, Lishui, Zhejiang, China

**Keywords:** metabolic syndrome, cardiovascular disease, elderly, major adverse cardiovascular events, mortality

## Abstract

**Background:**

Cardiovascular disease (CVD) remains a leading cause of morbidity and mortality globally, with metabolic syndrome (MS) being a significant contributor to major adverse cardiovascular events (MACE). While the relationship between MS and CVD is well established, limited studies have focused on elderly populations, particularly in the context of long-term cardiovascular outcomes. This study aims to fill this gap by investigating the impact of MS and its components on MACE in an elderly population.

**Methods:**

We conducted a prospective cohort study involving 3,352 elderly residents from the Northern Shanghai Study (NSS), followed for an average of 5.6 years. MS was defined based on modified NCEP ATP III criteria. The primary outcome was MACE, including non-fatal myocardial infarction (MI), non-fatal stroke, cardiovascular death, and all-cause mortality. Kaplan-Meier survival analysis and multivariate logistic regression were used to examine the association between MS and MACE.

**Results:**

MS was present in 44.2% of the cohort. Participants with MS had a significantly higher incidence of MACE compared to those without MS (12.69% vs. 9.30%, p=0.02). MS was confirmed as an independent risk factor for MACE (HR 1.471, 95% CI 1.195–1.181, p=0.001). Among the individual components of MS, the combination of central obesity, hypertension, and hyperglycemia (ABD+BP+GLU) showed the strongest predictive value for MACE (HR 3.001, 95% CI 1.640–5.492, p<0.001).

**Conclusions:**

MS is an independent predictor of MACE in elderly populations, with central obesity, hypertension, and hyperglycemia being the most critical risk factors. These findings underscore the importance of early identification and management of MS to reduce cardiovascular risk and improve long-term outcomes in elderly individuals.

## Graphical Abstract

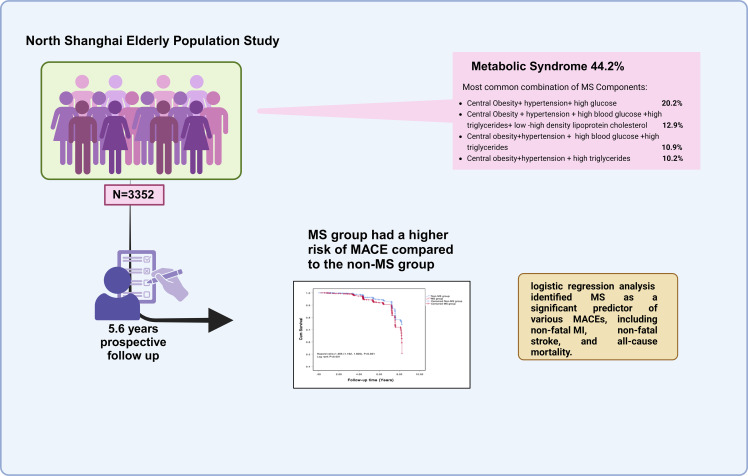

## 1 Introduction

Cardiovascular disease (CVD) remains a leading cause of morbidity and mortality worldwide, with its prevalence and associated disability increasing significantly over recent decades (1–3). The Global Burden of Disease study highlights a dramatic rise in CVD cases, reaching 525 million by 2019, nearly doubling from 1990 (1). As populations continue to age globally, CVD is expected to remain a major health burden, particularly in elderly populations (4). Major adverse cardiovascular events (MACE), including non-fatal myocardial infarction (MI), non-fatal stroke, and cardiovascular-related death, are key outcomes used to assess the severity of CVD and its impact on health (5, 6). Preventing these outcomes is crucial in managing cardiovascular risk, particularly among elderly individuals who are more susceptible to MACE due to the accumulation of risk factors with age.

Metabolic syndrome (MS) is defined by a cluster of metabolic abnormalities, including central obesity, hypertension, dyslipidemia, and impaired glucose metabolism (7, 8). Rising rates of MS, driven by increasing obesity and sedentary lifestyles, contribute significantly to adverse cardiovascular outcomes and mortality (9–11). Studies have consistently shown that MS is a major risk factor for CVD, significantly increasing the risk of MACE (12–14). Meta-analyses and large cohort studies have demonstrated that MS nearly doubles the likelihood of cardiovascular events, including MI and stroke (15–19). Given this elevated risk, MS is considered a pre-disease state, emphasizing the need for early intervention to prevent the progression to overt cardiovascular disease (20). Elderly individuals are particularly vulnerable to the effects of MS, as multiple cardiovascular risk factors tend to cluster and intensify with age, amplifying the risk of MACE. However, most studies have focused on general populations or younger individuals, with few research exploring the specific impact of MS on MACE in elderly populations particularly in rapidly urbanizing regions where the burden of CVD and MS is increasing. Understanding the relationship between MS and MACE in elderly is crucial for improving early identification of high-risk individuals and improving preventive care strategies.

The current study aims to address the research gap by assessing the association between MS and MACE in an elderly, community-dwelling population in northern Shanghai. Additionally, we examine the contribution of individual components of MS—such as central obesity, hypertension, dyslipidemia, and impaired glucose metabolism—to the risk of MACE. These insights will inform clinicians and public health policymakers to better target preventive measures and reduce cardiovascular risk in the elderly.

## 2 Materials and methods

### 2.1 Research subjects

The Northern Shanghai Study (NSS, ClinicalTrials.gov Identifier: NCT02368938) is a prospective study aimed at establishing cardiovascular risk scores based on a community-dwelling elderly Chinese population. The study seeks to identify relevant cardiovascular risk factors and target organ damage profiles to guide future interventions. From June 2014 to July 2019, a total of 3,590 residents from the northern Shanghai community were invited to participate in the study, with 3,363 individuals (93.7%) participating in the initial screening process. Among them, 3,352 participants (99.7%) completed an average follow-up of 2,048 days. This study was approved by the Ethics Committee of the Tenth People’s Hospital affiliated with Tongji University, and all participants provided written informed consent.

#### 2.1.1 Inclusion criteria

1. Age ≥ 65 years;
2. Community residents from northern Shanghai;
3. Ability to participate in long-term follow-up.

#### 2.1.2 Exclusion criteria

1. Severe heart disease (New York Heart Association Class IV) or end-stage renal disease (Chronic Kidney Disease Stage ≥ 4);
2. Malignant tumors with an expected survival of < 5 years;
3. History of stroke within the past 3 months.

All participants in this study were surveyed using a standard questionnaire, which included basic information such as gender, age, and family history of cardiovascular disease, as well as smoking history and status. During the interviews, previous diagnoses of diabetes, hypertension, coronary artery disease, stroke, and stroke types were also collected.

### 2.2 Research methodology

#### 2.2.1 Human body parameter measurement

Body weight is measured using a calibrated electronic scale with an accuracy of 0.1 kg, while height is measured using a tape measure. Body Mass Index (BMI) is calculated as follows: BMI = Weight (kg)/Height² (m²). Waist Circumference (WC) is measured as the horizontal circumference at the midpoint between the lower edge of the rib cage and the iliac crest along the midaxillary line (21–23). Blood Pressure (BP) measurement follows the recommendations of the European Society of Hypertension: the subject should sit quietly for more than 10 minutes. BP is measured 3 times using a conventional cuff mercury sphygmomanometer, with a 3-minute interval between each measurement, and the average value is taken.

#### 2.2.2 Laboratory tests

Blood samples were collected from study participants after a 10-hour overnight fasting period and analyzed by the Department of Laboratory Medicine at Shanghai Tenth People’s Hospital using standardized protocols and certified equipment. Fasting plasma glucose (FPG) was measured using the hexokinase method on an automated biochemical analyzer (Hitachi 7600), with intra- and inter-assay coefficients of variation (CVs) of 1.5% and 2.0%, respectively. Lipid profiles, including total cholesterol (TC), triglycerides (TG), high-density lipoprotein cholesterol (HDL-c), and low-density lipoprotein cholesterol (LDL-c), were determined using enzymatic colorimetric assays. TC and TG measurements had intra-assay CVs of 1.2% and 1.8% and inter-assay CVs of 1.5% and 2.1%, respectively. HDL-c was measured using a homogeneous enzymatic assay (intra-assay CV: 1.3%; inter-assay CV: 1.7%), while LDL-c was calculated using the Friedewald formula (for TG < 4.5 mmol/L) or directly measured (intra-assay CV: 1.4%; inter-assay CV: 1.9%). Internal quality control samples were analyzed daily, and the laboratory participated in external quality assurance programs to ensure accuracy and precision.

#### 2.2.3 Definition of metabolic syndrome

According to the Third Report of the National Cholesterol Education Program (NCEP) Adult Treatment Panel III (ATP III) in 2005 (24), MS is defined as the presence of at least three of the following metabolic disorders, with modifications made for the Asian population: Waist Circumference (WC) ≥90 cm for men and ≥80 cm for women; Blood Pressure (BP) ≥130/85 mmHg and/or treatment for previously diagnosed hypertension; Fasting Plasma Glucose (FPG) ≥5.6 mmol/L and/or previously diagnosed type 2 diabetes; Serum Triglycerides (TG) ≥1.7 mmol/L and/or specific treatment for lipid abnormalities; High-Density Lipoprotein Cholesterol (HDL-c) <1.03 mmol/L for men and <1.29 mmol/L for women and/or specific treatment for lipid abnormalities.

### 2.3 Follow-up

**Figure 1:**
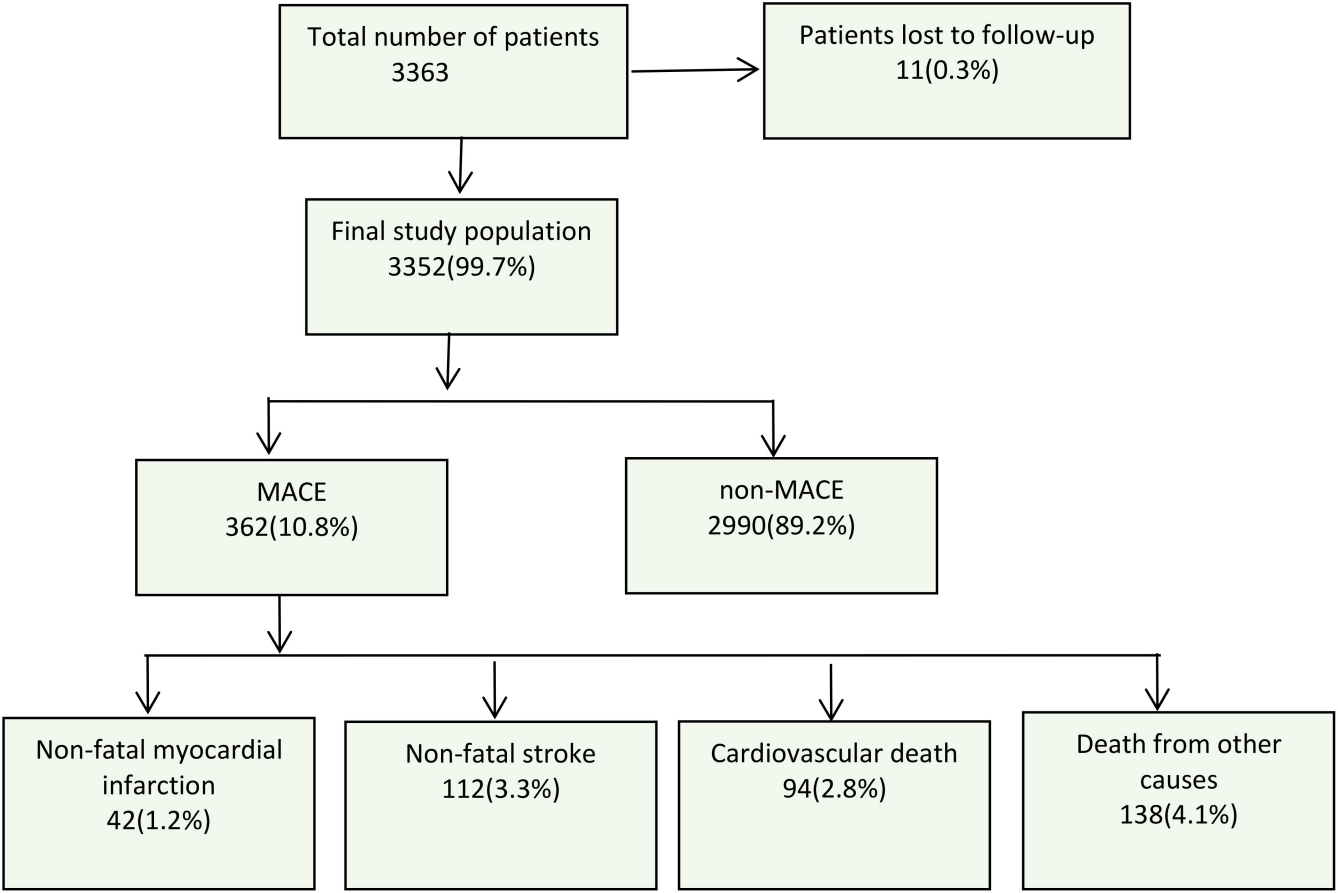
Flow chart of the study process and follow-up conditions. MACE, major adverse cardiovascular events.

All participants were followed up through telephone interviews or on-site visits. The follow-up period ranged from 3 to 8 years, with an average follow-up time of 5.6 years. During this period, new diagnoses of diseases were tracked through self-reports, including non-fatal myocardial infarction, non-fatal stroke, cardiovascular death, and all-cause mortality. The exact time of these events was recorded. In the case of multiple clinical events occurring in the same patient, the first event was considered the endpoint event. Patients who were lost to follow-up or without MACE were regarded as censored patients. The follow-up endpoint event data is shown in **Figure 1**.

### 2.4 Statistical analysis

Data were analyzed using SPSS 13.0, with p < 0.05 considered significant. Normality was assessed via the Kolmogorov-Smirnov test: normally distributed variables were reported as mean ± SD (compared via t-test), while non-normal data used median (IQR) (compared via Wilcoxon test). Categorical variables were expressed as n (%) (compared via χ² test). Univariate analysis screened risk factors for MACE, followed by Kaplan-Meier analysis (log-rank test) to assess MS-MACE associations over time. Logistic regression calculated ORs (95% CI) for MACE risk, and Cox proportional hazards models estimated adjusted HRs (95% CI) for MS and its components, with checks for proportional hazards assumptions.

## 3 Results

### 3.1 Clinical characteristics of study subjects

This study followed up on 3352 subjects, categorized into the MS group (1481 subjects, 44.2%) and the non-MS group (1871 subjects, 55.8%) according to NCEPIII. As shown in **Table 1**, there were no significant differences between the two groups in terms of age, systolic BP, TC, LDL-c, history of diabetes, and early onset of CVD. However, the MS group had higher waist circumference, hip circumference, waist-to-hip ratio (WHR), diastolic BP, TG, HDL-c, and FBG levels compared to the non-MS group (all P values <0.01). The MS group also had a higher prevalence of hypertension, stroke, MI, and a family history of hypertension. Interestingly, the proportion of smokers was higher in the non-MS group (all P values <0.01).

**Table 1:** Baseline clinical characteristics of the study subjects.

Variables	Total (3352)	MS (1481)	Non-MS (1871)	*P-value*
Age (years)	71. ± 6.0	71.51 ± 6.0	71.51 ± 6.0	0.353
Gender (male)	1460 (43.6%)	557 (37.6%)	905 (48.4%)	<0.001
BMI (kg/m^2^)	24.6 ± 3.6	26.1 ± 3.4	23.3 ± 3.2	<0.001
WC (cm)	86.6 ± 9.9	91.2 ± 8.6	83.0 ± 9.2	0.001
HC (cm)	96.6 ± 7.2	99.3 ± 6.9	94.5 ± 6.6	<0.001
W/H	0.90 ± 0.07	0.92 ± 0.06	0.88 ± 0.06	<0.001
SBP (mmHg)	135.8 ± 17.2	140.2 ± 16.2	132.3 ± 17.2	0.254
DBP (mmHg)	79.5 ± 9.7	80.9 ± 9.8	78.4 ± 9.4	<0.001
FPG (mmol/l)	5.78 ± 1.8	6.37 ± 2.01	5.31 ± 1.34	<0.001
TC (mmol/l)	5.10 ± 1.04	5.12 ± 1.12	5.10 ± 0.98	0.154
TG (mmol/l)	1.62 ± 1.04	2.13 ± 1.28	1. 22 ± 0.52	<0.001
HDL-c (mmol/l)	1.40 ± 0.37	1.22 ± 0.29	1.55 ± 0.36	<0.001
LDL-c (mmol/l)	3.13 ± 0.90	3.16 ± 0.98	3.11 ± 0.84	0.125
History of hypertension n (%)	1780 (52.1%)	1023 (69.1%)	757 (40.5%)	<0.001
History of diabetes n (%)	654 (19.5%)	478 (32.3%)	176 (9.4%)	0.061
Previous CVD n (%)	1070 (31.9%)	531 (35.9%)	537 (28.7%)	<0.001
Previous stroke history n (%)	627 (18.7%)	315 (21.3%)	312 (16.7%)	0.001
Family history of Hypertension n (%)	1768 (52.7%)	839 (56.7%)	929 (49.7%)	<0.001
Family history of CVD n (%)	719 (21.4%)	333 (22.5%)	386 (20.7%)	0.086
Smokers n (%)	830 (24.8%)	325 (21.9%)	505 (27.0%)	<0.001

BMI, body mass index; WC, waist circumference; HC, hip circumference; SBP, systolic blood pressure; DBP, diastolic blood pressure; FPG, fasting blood glucose; TC, total cholesterol; TG, triglycerides, HDL-c, high-density lipoprotein cholesterol; LDL-c, low-density lipoprotein cholesterol; CVD, cardiovascular disease; MS, metabolic syndrome.

Analysis of the 1481 subjects with metabolic syndrome revealed that the most common combination of metabolic syndrome components was central obesity + hypertension + high blood glucose (ABD+BP+GLU) (299 cases, 20.2%), followed by central obesity + hypertension + high blood glucose + high triglycerides + low high-density lipoprotein cholesterol (ABD+BP+GLU+TRI+HDL-c) (191 cases, 12.9%), central obesity + hypertension + high blood glucose + high triglycerides (ABD+BP+GLU+TRI) (162 cases, 10.9%), and central obesity + hypertension + high triglycerides (ABD+BP+TRI) (151 cases, 10.2%). There were gender differences in the combination’s ABD+BP+GLU+TRI+HDL-c (12.9%), ABD+BP+TRI+HDL-c (10.0%), ABD+GLU+TRI+HDL-c (14.7%), BP+GLU+TRI+HDL-c (2.8%), and BP+GLU+TRI (4.4%) (see **Table 2**).

**Table 2:** Combination of metabolic syndrome components in MS group.

Variables	Total (n=1481) n (%)	Male (n=557) n (%)	Female (n=924) n (%)	*P-value*
ABD+BP+GLU+TRI+HDL-c	191 (12.9%)	48 (8.6%)	143 (15.5%)	<0.001
ABD+BP+GLU+TRI	162 (10.9%)	62 (11.1%)	100 (10.8%)	0.971
ABD+BP+GLU+HDL-c	106 (7.2%)	31 (5.6%)	75 (8.1%)	0.640
ABD+BP+TRI+HDL-c	148 (10.0%)	38 (6.8%)	110(11.9%)	0.020
ABD+GLU+TRI+HDL-c	27 (1.8%)	5 (0.9%)	22 (2.4%)	0.044
ABD+BP+GLU	299 (20.2%)	125(22.4%)	177(19.2%)	0.351
ABD+BP+TRI	151 (10.2%)	60 (10.8%)	91 (9.8%)	0.571
ABD+BP+HDL-c	102 (6.9%)	34 (6.1%)	68 (7.4%)	0.209
ABD+GLU+TRI	17 (1.1%)	4 (0.7%)	13 (1.4%)	0.228
ABD+GLU+HDL-c	19 (1.3%)	8 (1.4%)	11 (1.2%)	0.684
ABD+TRI+HDL-c	41 (2.8%)	9 (1.6%)	32 (3.5%)	0.036
BP+GLU+TRI+HDL-c	42 (2.8%)	24 (4.3%)	18 (1.9%)	0.008
BP+GLU+TRI	56 (3.8%)	45 (8.1%)	11 (1.2%)	<0.001
BP+GLU+HDL-c	27 (1.8%)	15 (2.7%)	12 (1.3%)	0.052
BP+TRI+HDL-c	65 (4.4%)	31 (5.6%)	33 (3.6%)	0.057
GLU+TRI+HDL-c	10 (0.7%)	4 (0.7%)	6 (0.6%)	0.876

ABD, central obesity; BP, hypertension; GLU, high blood glucose; TRI, high triglycerides; HDL-c, low high-density lipoprotein cholesterol.

### 3.2 Analysis of endpoint events

The endpoint was MACE, including non-fatal MI, non-fatal stroke, cardiovascular death, and death from other causes. After an average follow-up of 5.6 years, there were a total of 362 (10.8%) endpoint events, including 41 (1.2%) cases of non-fatal MI, 112 (3.3%) cases of non-fatal stroke, 94 (2.8%) cases of cardiovascular death, and 138 (4.1%) cases of death from other causes (**Figure 1**).

At the end of the follow-up, the overall probability of endpoint events was higher in the MS group compared to the non-MS group, with a statistically significant difference between the two groups (188 cases, 12.69% vs. 174 cases, 9.30%, p=0.02). Regarding specific types of MACE, the MS group had higher rates than the non-MS group for non-fatal MI, non-fatal stroke, and overall mortality (2.02% vs. 1.64%, p<0.001; 4.04% vs. 2.77%, p=0.052; 7.94% vs. 6.13%, p<0.001). However, the difference in cardiovascular death between the two groups was not significant (3.43% vs. 2.34%, p=0.060) (**Table 3**).

**Table 3:** Comparison of endpoint events between two groups.

Variables	MS (1481)	non-MS (1871)	*P-value*
MACE	188 (12.65%)	174 (9.30%)	0.002
Non-fatal MI	30 (2.03%)	11 (0.59%)	<0.001
Non-fatal stroke	60 (4.06%)	52 (2.78%)	0.052
Cardiovascular death	54 (3.65%)	40 (2.14%)	0.060
All-cause death	118 (7.97%)	115 (6.15%)	<0.001

MACE, major adverse cardiovascular events; MI, myocardial infarction; MS, metabolic syndrome.

### 3.3 Regression analysis of endpoint events

As shown in **Table 4**, we analyzed the relationship between MS and various endpoint events. According to the NCEP III (2005) criteria for identifying study subjects with MS, the unadjusted logistic regression analysis showed the following MACE risks: overall MACE (OR = 1.416, 95% CI: 1.138–1.762, P = 0.02), non-fatal myocardial infarction (OR = 3.200, 95% CI: 1.633–6.172, P = 0.01), non-fatal stroke (OR = 1.475, 95% CI: 1.011–2.125, P = 0.044), cardiovascular death (OR = 1.582, 95% CI: 0.938–2.861, P = 0.088), and all-cause mortality (OR = 1.320, 95% CI: 1.011–1.722, P = 0.041). After adjusting for age, gender, smoking history, family history of hypertension, and family history of early-onset CVD, the results remained similar. MS was significantly associated with overall endpoint events, non-fatal myocardial infarction, non-fatal stroke, and all-cause mortality (all P < 0.05).

**Table 4:** Logistic regression analysis of the association between MS and endpoint events.

Variables	OR(95% CI)			
	Modle1	Modle2	Modle3	Modle4
MACE	1.416 (1.138–1.762) *	1.525 (1.213–1.916) *	1.522 (1.211–1.913) *	1.520 (1.209–1.912)*
Non-fatal-MI	3.200 (1.633–6.172) *	3.567 (1.812–7.021) *	3.549 (1.802–6.988) *	3.539 (1.759–6.976)*
Non-fatal-Stroke	1.475 (1.011–2.125) *	1.507 (1.030–2.206) *	1.505 (1.029–2.203) *	1.493 (1.019–2.187)*
CVD death	1.320 (1.011–1.722)	1.620 (0.936–2.803)	1.426 (1.0075–1.892)	1.434 (1.080–1.904)
All-cause death	1.582 (0.933-2.681)*	1.426 (1.075-1.892) *	1.656 (0.954-2.875) *	1.619 (0.926-2.830)*

Model 1, unadjusted; Model 2, adjusted for age and gender; Model 3, adjusted for age, gender, and smoking history; Model 4, adjusted for age, gender, smoking history, family history of hypertension, and family history of early-onset CVD. *P <0.05. MACE, major adverse cardiovascular events; MI, myocardial infarction; CVD, cardiovascular disease.

**Figure 2:**
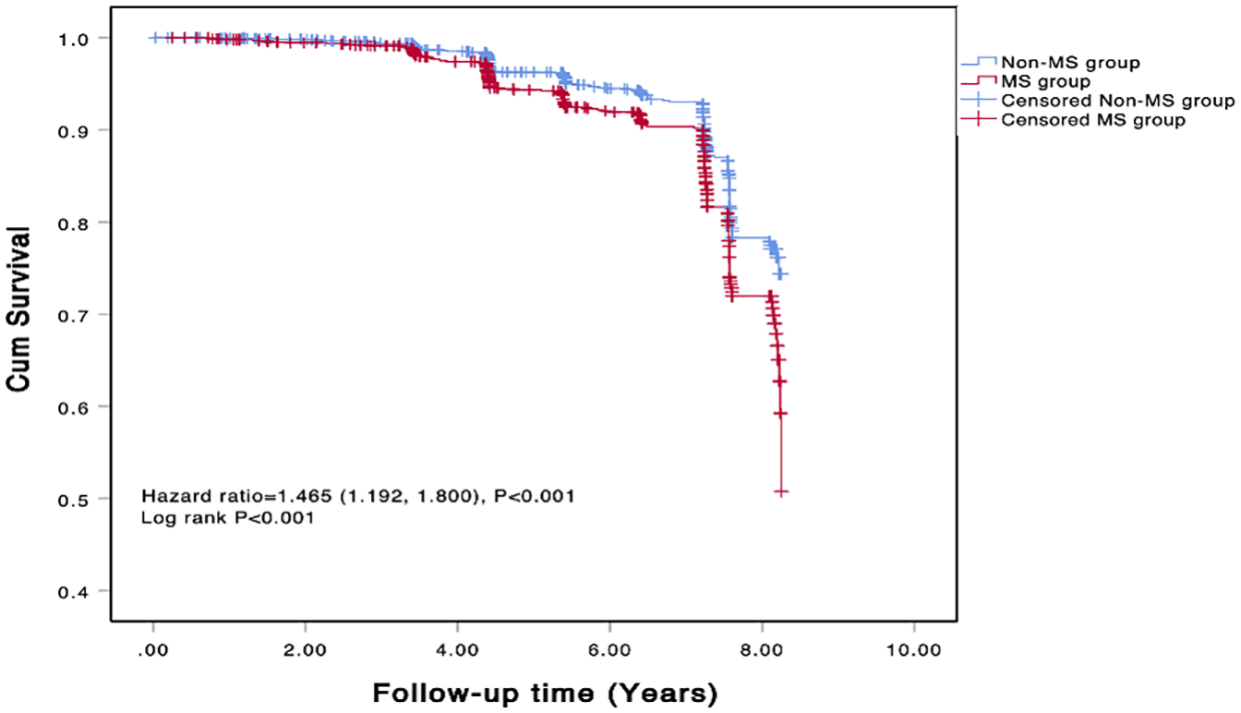
Kaplan-Meier survival curves of MACE. MS, metabolic syndrome.

The COX regression analysis indicated that MS is an independent risk factor for MACE [HR 1.471, 95% CI 1.195-1.181, p=0.001], primarily due to the increased risk of all-cause mortality, non-fatal stroke, and non-fatal MI (HR 1.422, 95% CI 1.097-1.843, p=0.008; HR 1.506, 95% CI 1.039-2.184, p=0.031; HR 3.484, 95% CI 1.780-6.811, p<0.001) (**Table 5**). There was a significant difference in the Kaplan-Meier curves between the two groups (p=0.001), showing similar results that the high MS group had a higher risk of MACE compared to the non-MS group (**Figure 2**).

**Table 5:** Multivariate COX regression analysis of MS and various adverse events.

Variables	HR (95% CI)	*P*
MACE	1.471 (1.195-1.181)	0.001
Non-fatal MI	3.484 (1.780-6.811)	<0.001
Non-fatal stroke	1.506 (1.039-2.184)	0.031
Cardiovascular death	1.325 (0.874-2.010)	0.186
All-cause death	1.422 (1.097-1.843)	0.008

MACE, major adverse cardiovascular events; MI, myocardial infarction.

**Figure 3:**
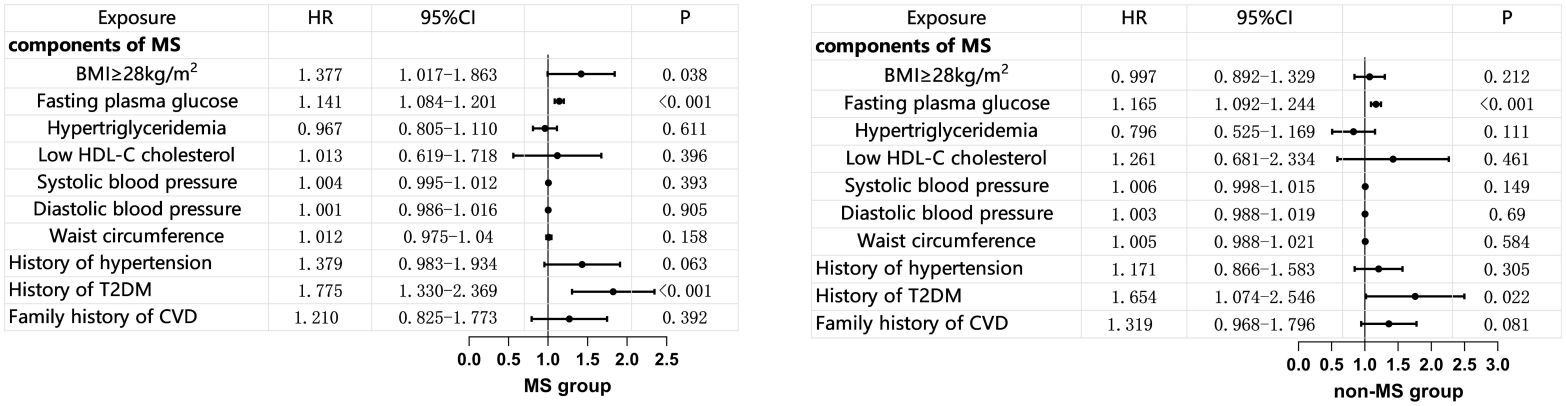
Multivariate COX Regression Analysis of MS Components and MACE (adjusted for age, gender, and smoking status). BMI, body mass index; HDL-C, high density lipoprotein-C; T2DM, Type 2 Diabetes Mellitus; CVD, cardiovascular disease; MS, metabolic syndrome.

Within the MS group, multivariate COX regression analysis showed that a BMI ≥ 28 mg/m² is an independent risk factor for MACE (HR 1.377, 95% CI 1.017–1.863, p=0.038). FBG was an independent risk factor for MACE in both the MS group and the non-MS group (HR 1.414, 95% CI 1.084–1.201, p<0.001; HR 1.165, 95% CI 1.092–1.204, p<0.001), while other components of MS did not show an increased risk for MACE (**Figure 3**).

Multivariate COX regression analysis of four common MS components with MACE showed that ABD+BP+GLU had a higher predictive value for MACE, with statistical significance compared to the control group (HR 3.001, 95% CI 1.640-5.492, p<0.001) (**Table 6**).

**Table 6:** COX regression analysis of different metabolic syndrome states and MACE (adjusted for age, gender, and smoking history).

MS status	MACE		
	N	HR (95%CI)	P
MS component 0 (control)	253 (14)		
ABD BP GLU TRI HDL-c	191 (24)	2.748 (1.362-5.562)	0.005
ABD BP GLU	299 (50)	3.001 (1.640-5.492)	<0.001
ABD BP GLU TRI	162 (34)	2.809 (1.395-5.659)	0.004
ABD BP TRI	151 (13)	1.812 (0.850-3.872)	0.125

ABD, central obesity; BP, hypertension; GLU, high blood glucose; TRI, high triglycerides; HDL-c, low high-density lipoprotein cholesterol; MACE, major adverse cardiovascular events.

## 4 Discussion

The present study aimed to explore the clinical characteristics and the relationship between MS and MACE in an elderly population from the Northern Shanghai Study, a prospective cohort that followed 3,352 subjects over an average of 5.6 years. The findings from this cohort indicate that MS significantly increases the risk of MACE, including non-fatal MI, non-fatal stroke, cardiovascular death, and all-cause mortality, confirming MS as a critical determinant of cardiovascular health in elderly individuals.

The analysis revealed that subjects with MS had notably higher waist circumference, hip circumference, waist-to-hip ratio, diastolic BP, TG, HDL-c, and FBG levels compared to their non-MS counterparts. This is consistent with previous studies that associate MS with metabolic abnormalities, further emphasizing that MS represents a cluster of conditions driving cardiovascular risk. Additionally, the prevalence of hypertension, stroke, MI, and a family history of hypertension were significantly higher in the MS group, further highlighting the burden of MS on cardiovascular health. Interestingly, the non-MS group had a higher proportion of smokers, which might indicate that factors other than smoking contribute significantly to the cardiovascular risk in the MS group.

Our findings emphasize that central obesity is a crucial component of MS among the elderly population. Specifically, four common combinations of MS components centered around central obesity were identified: ABD+BP+GLU, ABD+BP+GLU+TRI+HDL-c, ABD+BP+GLU+TRI, ABD+BP+TRI. These combinations accounted for 54% of the total MS cases. Previous research supports our findings regarding the prevalence and impact of central obesity (25–27). The Amsterdam Longitudinal Study on Aging (LASA) demonstrated that over a three-year follow-up period, BMI served as an independent predictor of metabolic syndrome in community-dwelling adults aged 55-85 years (28, 29). Additionally, Chu-Sheng Lin’s retrospective cohort study utilizing the New Taipei City Elderly Health Examination Database (NTCHD) from 2014 to 2016 observed an increase in the prevalence of metabolic syndrome and central obesity, alongside a decrease in BMI after a two-year follow-up period (30). Thus, aligns with previous studies, our findings also show that central obesity is a predominant feature of metabolic syndrome in the elderly population. However, while central obesity and hypertension were highlighted as key drivers of cardiovascular risk in our study, the role of dyslipidemia, particularly low HDL-c and elevated triglycerides, warrants further exploration. Although hypertriglyceridemia was not identified as an independent risk factor for MACE in our cohort, its interplay with other MS components, such as central obesity and insulin resistance, may still contribute to cardiovascular risk. For example, low HDL-c levels, which are a hallmark of dyslipidemia in MS, have been linked to oxidative stress and inflammation, as evidenced by the negative correlation between total antioxidant status and HDL-c in elderly individuals with MS (31). This suggests that oxidative stress may mediate the relationship between dyslipidemia and cardiovascular risk. In our study, HDL-c levels were significantly lower in the MS group compared to the non-MS group, consistent with the metabolic abnormalities associated with MS. Future studies should investigate the mechanisms underlying these associations, including the role of oxidative stress and inflammatory markers, to better understand their implications for cardiovascular risk.

The data from our cohort shows that the incidence of MACE was higher in individuals with MS compared to those without MS. Specifically, the MS group exhibited higher rates of non-fatal MI, non-fatal stroke, and overall mortality. These results are consistent with previous research demonstrating that individuals with MS are at a significantly higher risk of experiencing cardiovascular events. For example, a meta-analysis by Kazlauskiene et al. found that individuals with MS had a twofold increased risk of cardiovascular events, such as MI and stroke, compared to individuals without MS (32). Notably, non-fatal MI emerged as a major outcome, with individuals with MS facing a threefold increased risk. This aligns with prior studies that have demonstrated a strong link between MS and coronary artery disease (33–35). In a study by Sedaghat et al. MS is associated with a higher risk of MI, particularly in individuals with excess body weight. A meta-analysis found that individuals with obesity and MS had a HR of 1.68 for MI, indicating a significant positive association (36). The elevated risk of non-fatal MI in our study suggests that the combination of metabolic abnormalities in MS, particularly central obesity and dyslipidemia, may contribute to the development of atherosclerosis and coronary artery disease, ultimately leading to myocardial infarction. Similarly, the increased risk of non-fatal stroke in our study population supports previous research linking MS to cerebrovascular events (37–39). A meta-analysis of prospective cohort studies conducted by Li et al. found that individuals with MS have a 70% higher risk of incident stroke compared to those without MS, with RR of 1.70 (40). Our study found a similar relationship, with MS significantly increasing the likelihood of non-fatal stroke. This relationship may be driven by the hypertensive and atherogenic effects of MS components, such as elevated blood pressure and dyslipidemia, which are well-known risk factors for stroke. The increased risk of stroke in individuals with MS highlights the importance of managing these metabolic abnormalities to prevent cerebrovascular events, particularly in elderly individuals who are more vulnerable to stroke.

Interestingly, while cardiovascular death did not reach statistical significance between the MS and non-MS groups in our study, the trend toward higher cardiovascular mortality in the MS group is consistent with prior research. Several studies have reported a modest but significant association between MS and cardiovascular death (14, 41, 42). For instance, a study by Dekker et al. reported that MS increases the risk of cardiovascular death by 2-fold (43). The lack of statistical significance in our study may be due to the relatively small number of cardiovascular deaths during the follow-up period, but the observed trend is in line with the broader literature. One of the more striking outcomes in our study was the significantly higher all-cause mortality rate in individuals with MS compared to those without. This aligns with the results of previous studies, such as a study involving a large cohort from the LIPIDOGRAM studies found that MS significantly increased mortality risk in both obese and non-obese individuals, with HR ranging from 1.7 to 2.11 depending on the criteria used for MS diagnosis (44, 45). The elevated mortality risk in individuals with MS can be attributed to the combined effect of multiple metabolic abnormalities, which contribute not only to cardiovascular diseases but also to other chronic conditions, such as type 2 diabetes and cancer, further increasing overall mortality.

The logistic regression analysis identified MS as a significant predictor of various MACEs, including non-fatal MI, non-fatal stroke, and all-cause mortality. These associations persisted even after adjusting for potential confounders such as age, gender, smoking history, and family history of hypertension and early-onset CVD. Further supporting these findings, the Cox regression analysis indicated that MS is an independent risk factor for MACE, primarily driven by an increased risk of all-cause mortality, non-fatal stroke, and non-fatal MI. Notably, within the MS group, BMI of ≥28 mg/m² emerged as an independent risk factor for MACE, underscoring the significant role of obesity in cardiovascular risk. The association between higher BMI and increased cardiovascular risk observed in our study is supported by previous research, including the INTERHEART study, which identified abdominal obesity as a key contributor to MI risk (46, 47). Our findings emphasize the importance of weight management in elderly individuals with MS to reduce cardiovascular risk. In contrast, among those without metabolic syndrome, a BMI of ≥28 mg/m² did not predict the risk of MACE effectively. This discrepancy may be attributed to the lower average BMI of the non-MS group in this study (mean BMI of 23.3 mg/m²). Therefore, when considering whether BMI can predict MACE, the characteristics of the population should be considered. Another set of data from this study showed that participants with a high middle-upper arm circumference (MUAC) were associated with higher body weight, BMI, WC, and WHR, indicating more obesity and central obesity. However, among participants with a high MUAC, there was a cardiovascular risk benefit (48). This phenomenon might be related to the pattern of fat accumulation, which warrants further investigation. Additionally, among the individual components of MS, FBG was found to be an independent risk factor for MACE in both the MS and non-MS groups. This finding is consistent with numerous studies showing that hyperglycemia, even in the prediabetic range, is strongly associated with increased cardiovascular risk (49, 50). The role of hyperglycemia in promoting endothelial dysfunction, inflammation, and atherosclerosis likely explains its contribution to cardiovascular events in our cohort, underscoring the critical impact of glycemic control on cardiovascular outcomes.

The multivariate Cox regression analysis of common MS components revealed that the combination of central obesity, hypertension, and high blood glucose (ABD+BP+GLU) had a higher predictive value for MACE. This combination significantly increased the risk of MACE compared to the control group, highlighting the importance of targeting these specific components in the management of MS and underscores the necessity of targeting central obesity and its associated metabolic abnormalities in the prevention and management of cardiovascular risk among elderly individuals with MS. Interestingly, hypertriglyceridemia was not identified as a risk factor for cardiovascular risk; in fact, higher triglyceride levels in the non-MS group appeared to be a protective factor for cardiovascular health. There has been ongoing debate about whether lowering triglycerides can provide cardiovascular benefits (51, 52). Some research has confirmed that triglycerides are an independent risk factor for cardiovascular disease (53–55). However, the results of the PROMINENT study (a multicenter, double-blind, randomized controlled trial) released at the 2022 American Heart Association (AHA) annual meeting were similar to our findings. This study showed that patients with mild to moderate hypertriglyceridemia who received fibrate therapy did not have a lower incidence of cardiovascular events compared to the placebo group (56). This phenomenon may be related to the increased efficiency of converting triglyceride-rich lipoprotein remnants into low-density lipoprotein rather than an increase in clearance efficiency by the liver. This suggests that the relationship between triglycerides and cardiovascular risk may be more complex than previously understood, potentially influenced by factors like lipid metabolism efficiency and further research is needed to elucidate the mechanisms involved.

This study has several limitations that should be acknowledged. First, the cohort was composed of elderly individuals from a specific community in northern Shanghai, which may limit the generalizability of the findings to other geographic regions, ethnic groups, or younger populations. The unique demographic, cultural, and environmental characteristics of this population may not be representative of other settings, potentially influencing the applicability of the results. Second, the absence of repeated measurements of anthropometric and biological parameters throughout the follow-up period restricts our ability to capture changes in participants’ health status that could impact cardiovascular outcomes. For example, fluctuations in blood pressure, lipid levels, or glucose metabolism over time may contribute to cardiovascular risk, but these dynamics could not be assessed due to the lack of longitudinal data. Future studies should aim to include more diverse populations and incorporate repeated measurements at regular intervals to better understand the temporal relationships between risk factors and cardiovascular outcomes. Third, although our analysis adjusted for several confounders, such as age, gender, and smoking history, unmeasured variables like lifestyle factors (e.g., physical activity, dietary habits) and medication use (e.g., statins, antihypertensives) may influence the results. While some baseline data on the use of antihypertensive, lipid-lowering, and glucose-lowering agents were available, we lacked comprehensive information on changes in medication regimens, adherence, or dosages throughout the follow-up period. These pharmacological interventions can significantly impact metabolic parameters and cardiovascular outcomes, potentially modifying the relationship between metabolic syndrome and MACE. The absence of detailed, longitudinal data on these variables may have introduced residual confounding. Future studies should aim to incorporate more granular tracking of lifestyle and medication-related factors to enhance the accuracy and interpretability of findings. Fourth, the use of self-reported data for MACE may introduce recall bias, as participants may inaccurately remember or report events such as non-fatal MI or stroke. Although we cross-verified self-reported events with medical records whenever possible, the potential for misclassification remains. Fifth, the relatively small number of cardiovascular deaths in our cohort may limit the robustness of some conclusions, particularly regarding the association between MS and cardiovascular mortality. While the trend toward higher cardiovascular mortality in the MS group aligns with prior research, the lack of statistical significance may reflect insufficient power due to the low event rate. Finally, while our study highlights the individual contributions of MS components to cardiovascular outcomes, it does not fully explore the potential interactions and synergies among these components, such as how central obesity, insulin resistance, hypertension, and dyslipidemia may collectively amplify cardiovascular risk. Future studies should aim to include more diverse populations, incorporate repeated measurements, investigate interactions among MS components, and use more objective measures, such as hospital records or adjudicated endpoints, to reduce bias and improve the robustness of the findings. Despite these limitations, our study provides valuable insights into the cardiovascular health of elderly individuals in northern Shanghai, using standardized and rigorous laboratory methods to ensure the reliability of the findings.

In conclusion, this study confirms the strong association between MS and MACE in the elderly community of northern Shanghai. Our findings highlight that MS, particularly the combination of central obesity, hypertension, and high blood glucose, plays a pivotal role in predicting cardiovascular outcomes, including non-fatal MI, non-fatal stroke, and all-cause mortality. Given the increasing prevalence of MS in aging populations, these results underscore the urgency of targeted interventions to mitigate cardiovascular risk in elderly individuals.

Future research should focus on elucidating the mechanisms linking MS components to cardiovascular outcomes, particularly the role of oxidative stress, inflammation, and metabolic dysregulation. Longitudinal studies with repeated biochemical and anthropometric measurements are necessary to assess the temporal progression of MS and its impact on cardiovascular health. Additionally, exploring the effects of lifestyle modifications, such as diet and physical activity, as well as pharmacological interventions in MS management, will provide valuable insights into potential strategies for reducing MACE risk.

From a clinical perspective, our findings emphasize the need for early identification and comprehensive management of MS components, particularly central obesity and dysglycemia, to improve cardiovascular outcomes in elderly populations. Integrating routine MS screening into primary care settings, alongside personalized lifestyle and medical interventions, may help prevent the development of severe cardiovascular complications. Future studies should also evaluate the efficacy of targeted prevention programs in high-risk populations to optimize cardiovascular risk management in aging communities.

## Data Availability

The raw data supporting the conclusions of this article will be made available by the authors, without undue reservation.
